# Rimedii Individualizzati Del Dottor Tommaso Cigliano

**Published:** 1887-12

**Authors:** 


### BOOK NOTICES.

Rimedii Individualizzati per Sintomi e Malattie Ovvero, Grande Repertorio Clinico Omiopatico Del Dottor Tommaso Cigliano. Prezzo Lire 20; pp. 962. Napoli: 1887.

Our first regret, after looking over this grand clinical repertory, was that it is not published in English, that it might be accessible to English and American physicians. For it is a large and admirably arranged repertory that Dottor Cigliano has edited. All the leading works on therapeutics and practice have been consulted in its composition.

The repertory is alphabetically arranged; the symptom in general is first given, then the conditions, concomitants, sequelæ, etc. Altogether, the arrangement is very good and the matter thoroughly well edited. As an example, let us take the first part considered, the *Acromion.* Under it we have given six symptoms; thus: burning, heat, pain (subdivided into paralytic, rheumatic, etc.), lancinating, sensibility (also subdivided), etc. The next part is the abdomen, under which about one hundred different headings are given. By the use of heavy-faced type and running headings at top of each page, the use of the repertory is greatly facilitated. We can only compliment Dottor Cigliano on his great work, and again express a desire for an edition accessible to the English readers, who form such a great majority in the ranks of Hahnemann's followers.

